# Some Thoughts on Potentization and Dose

**Published:** 1892-09

**Authors:** A. R. Morgan

**Affiliations:** New York; Bureau of Homœopathic Philosophy, I. H. A.


### SOME THOUGHTS UPON POTENTIZATION AND DOSE.

A. R. Morgan, M. D., New York.

(Bureau of Homœopathic Philosophy, I. H. A.)

Among those who know no better the impression exists that about the only difference there is between homœopathic physicians is that one habitually resorts to comparatively strong and appreciable doses of medicine, while the other administers only tasteless pellets.

They have no idea that upon the question of dose hinges much of the philosophy of the practitioner; that his attitude upon this subject indicates quite conclusively whether he accepts the dynamic theory of the author of Homœopathy or still clings to the Jewish dispensation of medicine.

The old school of medicine regards the doctrine of the vital forces as an exploded theological dogma, a childish superstition, something incapable of physical demonstration, and, therefore, unworthy of serious consideration.

The medical agnostic fails to comprehend that his protoplasmic germs, his biological units are but the vehicles through which the life-forces of the Creator are manifested.

The school of vitalism recognizes a world of causes prior to the world of effects; it holds that matter and force are indestructible; that although endless changes may occur, no particle of matter can ever cease to exist, no unit force be lost. It holds that the "laws of nature" are but different modes of the Divine activity, that the material world is governed by prior forces, name these forces how you may, attraction, gravitation, electricity, magnetism, galvanism, chemical affinity or physical law, *vis medicatrix naturæ*, dynamic or spiritual forces or combine all under one supreme head and source of power—God.

Hahnemann was a representative thinker of the school of vitalism, and, up to the announcement of his revolutionary doctrines, occupied high rank among the eminent medical men of his time, and was a highly esteemed contributor to the standard medical periodicals of the day.

Becoming dissatisfied with the notorious uncertainties of the prevailing methods of practice, yet believing confidently, as he tells us, that "The Author of all good when He allowed diseases to injure His offspring, must have laid down a means by which their torments might be lessened or removed," he retired from active practice, determined to devote all the powers of his mind to study and reflection, resolved not to cease his efforts until he had arrived at definite and satisfactory conclusions.

Being a thorough and accomplished linguist, proficient in eight different languages—covering the medical literature of the civilized world—he was admirably equipped for the task he had undertaken. Scattered down the long line of empirical observation, denominated medical history, he found among many contrary records frequent reference made to instances where diseases had been cured by remedies capable of producing similar complaints, and he cites in detail hundreds of such cases so cured, and states distinctly that his object in referring to them "is to show that the art of curing homœopathically might have been discovered before my (his) time." (See introduction to *Organon*, Stratton's Edition, par. 45.)

Beside these references to the casual observations of former physicians, he distinctly points out the fact that transitory glimpses of the "law of similars" had more than once been caught by physicians prior to his time.

On page 75 (introduction to *The Organon*), he says: "Occasionally there have been certain physicians who *guessed* that medicines might cure diseases by the faculty which they possess of exciting morbid symptoms that resembled the disease itself," and then in a foot-note he adds: "In citing the following passages of writers * * * I wish to free myself from a reproach of having passed them over in silence in order to arrogate to myself the merit of the discovery."

On page 76 he states with complete frankness and generosity that "The Danish physician, Stahl, has above all writers expressed his conviction on this head most unequivocally," and he proceeds to quote from Stahl as follows: "The received method in medicine of treating diseases by opposite remedies—that is to say, by medicines which are opposed to the effects they produce (*contraria contrario*)—is completely false and absurd. * * * I am convinced, on the contrary, that diseases are subdued by agents which produce a similar affection (*similia similibus*)."

Thus it may be seen that the attention of our author was early in his career of investigation attracted to the significant relations which, at least sometimes, have been observed to exist between certain well-known remedies and their power to excite similar maladies; and at this stage, by a commanding stroke of genius, he conceived the idea of settling the controversy by ascertaining the complete and pure effects of drugs, by testing them upon the healthy subject.

That Hahnemann originated the idea of the proving of drugs is disputed by the old school. In an article entitled "Medicine" in the last (ninth) edition of the *British Encyclopædia* may be found the following statement: "Hahnemann carried out the proving of drugs upon the healthy to great elaboration, but he did not originate the idea."

In this article it is claimed that Haller, the great Swiss, and Tomassini, the distinguished Italian physician, both prior to Hahnemann, did something in this field.

However the facts may be we have no present opportunity of determining, but we can justly remark, if any degree of priority be claimed for others in this direction, that the prior claimants made little or no impression upon the dominant school of medicine, for the very obvious reason that without any definite system of practice, without any distinct and positive method of cure, such provings could be of no use, and were therefore abandoned, leaving little or no trace behind. Hahnemann began his investigations with a definite object in view, and after thorough, patient, intelligent, systematic study and comparison, after many tedious, prolonged, and self-sacrificing ordeals, at last succeeded in reducing the fragmentary observations of his predecessors to order, and thus by inductive philosophy was enabled to work out and announce to the world the existence of an heretofore *unsuspected universal* law of cure—not an occasional means of cure as was anticipated by Stahl and others, but an universal natural law of cure.

Thorough proving of drugs upon the healthy, therefore, was practically a new departure in medicine, and in fact constituted the first of the three grand steps which culminated in the evolution of a new, rational, and scientific system of medical practice.

Full recognition of the law of similars speedily succeeded the proving of drugs, and was the second radical step in the evolution of Homœopathy.

The third step and crowning achievement of our author was the discovery of *potentization*. Here Hahnemann has no rival claimant, no competitor. He began his experiments in this comparatively new and untried field of Homœopathy by adhering to traditional doses of medicine, but soon found that the sick whom he attempted to cure often grew worse under the new method of treatment. Shrewdly suspecting that the doses used were too large, he set about trying medicines moderately reduced in strength—*i. e.*, diluted.

On continuing his experiments in this direction, he found to his great amazement that the curative powers of drugs bore no proportionate relation to crude quantity, but under his peculiar mode of manipulation many drugs in common use, and many substances heretofore regarded as actually inert in their crude states, became invested with new and unsuspected activities and powers.

Our old friend Dr. Hering used sometimes in his talks to students to make a striking and beautiful comparison between Columbus and Hahnemann (we recall the idea if not the language).

Columbus, with no adequate conception of the vast field before him, set out to find a new path to the Indies. He took an exactly opposite course from that pursued by all former navigators, he sailed off into the unknown west, and was rewarded by the discovery of a continent, a consummation unsought for, undreamed of, but its importance was a thousand-fold greater than if he had accomplished his original design.

So with Hahnemann, who set out to reduce his doses and discovered *potentization*, an entirely new principle in posology, a whole continent in the world of therapeutics; a discovery of such transcendent importance as to be sufficient *alone* to immortalize its discoverer; a principle inseparably connected with advanced and intelligent Homœopathy; a principle which correlates the dynamic forces of the lower with the life-forces of the higher spirital kingdom of nature; a principle without which it is absolutely impossible to account rationally for the action of highly potentized drugs.

*To Hahnemann alone is due imperishable honor and renown for discovering, first, the existence of an universal law of cure; and, second, that the specific properties of drugs could be developed, transmitted, and utilized by potentization.*

Those who reject the idea of potentization, halt at the stage where Hahnemann began his first practical tests of the newly-discovered general law of cure.

Crude drugs have three grades of action, viz.:

Mechanical, chemical, and dynamical. The first two are of comparatively little importance to homœopathicians. This is shown by our obtaining our best provings from drugs more or less highly potentized—that is, from thirtieth upward—and the fact that we obtain more ample provings from dynamized than crude preparations shows the folly of those who expect to obtain exhaustive provings from crude drugs alone.

We claim that the full power of a drug—*i. e.*, its dynamic quality—is hampered and restrained by its grosser material elements, by its very crudity; we claim that entirely new activities are developed and liberated by potentization, as may be seen by the development of active characteristic properties—*i. e.*, substances, completely inert in their crude state.

We can understand why quantity becomes an indispensable factor to the materialist who confines his practice to appreciable doses of crude drugs.

We can understand the line of reasoning which predicates coarseness and fineness of particles pulverized within certain reasonable limitation by the trituration, for instance, of silex, salt, sugar; we can comprehend how the particles of these and similar solid substances may be ground finer and finer quite indefinitely because their particles are held together by what is called cohesive attraction, and a uniform diffusion or blending of molecules cannot take place between solid bodies. But it is quite different with solutions. The crystals of common salt or sugar may be dissolved in water and become amorphous liquids that are so uniformly diffused as to completely destroy the existence of coarser or finer particles.

The process of attenuation by the Hahnemannian method may be continued until all trace of quantity has vanished beyond the recognition of every possible physical test, yet we find dynamic quality or *potentiality* surviving.

The careful observer, upon testing their attenuation upon the animal body, finds in the life-force of the organism an apparatus far more delicate and sensitive than is afforded by any of our mere physical instruments.

Here we enter the mysterious realm of imponderable forces and encounter a problem beyond the reach of human analysis—a region where our conceptions are limited to observation of effects alone.

As we have stated elsewhere, all that we know, all that we can ever hope to know of these elemental forces we gather from a study of their visible and sensible effects.

It is so with all our knowledge of medicinal action, whether that of the crude or protentized drug.

Precisely the same line of evidence is available in experiments with either or both. We simply record our observations. We administer the potentized drug in accordance with the rules laid down in *The Organon*, and get unmistakable curative results; the scepticism of those who do not follow *the rules*, and therefore do not obtain like results, to the contrary notwithstanding.

Practitioners are generally known as "high" or "low" potency advocates, and it is a significant fact that the advocates of the low potencies exclusively almost universally reject the dynamic theory of Hahnemann, while, on the other side, those who advocate the use of the high potencies generally accept the dynamic philosophy.

It is not strange to me that the materialist is either an allopathist or an eclectic, for he could not be otherwise and be consistent. I honestly think his sense and logic more sound than that of the professed homœopathist, who, while scouting the idea of a purely dynamic action in drugs, still admits that he has seen curative results from any remedy carried above the twelfth potency. Once admit the efficiency or even the possibility of medicinal action in drugs carried to the twelfth potency or above, where no possible physical test reveals the presence of the crude drug, and the material hypothesis is incapable of demonstration, and therefore falls to the ground.

The practice of those who limit themselves to the use of the low potencies only is almost sure to degenerate into a sort of eclecticism, into a recourse to palliative measures, into the use of the hypodermic syringe, and more or less crude drugs. Then people sometimes, while claiming still to be homœopathists, seem to pride themselves on sending their deluded patients to the common drug store for crude compound prescriptions, the effect of which they and everybody else are lamentably ignorant.

It is useless to attempt to build up a sound and loyal homœopathician out of such timber; it is spongy with erroneous ideas and honey-combed with false pretenses.

Entire unanimity upon the subject of dose, however, does not exist among the radical homœopathicians.

There are those who assert that when the higher potencies fail (no matter how high) to produce curative results, it is useless to try the lower ones; others insist upon the right of private judgment as to what really constitutes the minimum dose in any given case of sickness.

Exactly what does constitute the minimum dose it is not easy to determine, and while we have many wise suggestions, it must be admitted that as yet we have no absolute and inflexible rule to guide us.

Hahnemann says "It often happens from various causes, which at all times cannot be discovered that even large doses of homœopathic medicines effect a cure, without causing any notable injury."

We know that may old school physicians have been converted to Homœopathy by observing that some of their most successful cures have been brought about by crude remedies used, unintentionally, in compliance with our law of similars.

Hahnemann cites hundreds of illustrations from old school practice, where cures were brought about by what J. J. Garth Wilkinson happily denominates "*the latency of Homœopathy in the common sense of his predecessors.*" (Introduction to *The Organon.*)

Hahnemann, in discussing the problem, "How far the dose of a homœopathic remedy in any given case of disease ought to be reduced in order to derive from it the best possible cure," says, "It may be readily conceived that *no theoretical conjecture will furnish an answer to this problem*, and that it is not by such means we can establish, in respect to each individual medicine, the quantity of the dose that suffices to produce the homœopathic effect, and accomplish a prompt and gentle cure. No reasoning, however ingenious, will avail in this instance. It is by pure experiments only and precise observations that this object can be attained." (*The Organon*, § 278.)

In the further discussion of this subject, Hahnemann says, "It has been fully proved by pure experiments *that when a disease does not evidently depend upon the impaired state of an important organ*, even though it were of a chronic nature and complicated, and due care has been taken to remove from the patient all foreign medicinal influences, the dose of the homœopathic remedy can never be sufficiently small so as to be inferior to the power of the natural disease which it can at least partially extinguish and cure, *provided* it be capable of producing only a small increase of symptoms immediately after it is administered." (§ 279, *Organon.*)

From this it is evident that Hahnemann thought the dose might be made too small where the disease depended upon an impaired state of an important organ, and this view is somewhat confirmed by the latitude of dose recommended in § 280, viz.: "This incontrovertible axiom, founded upon experience, will serve as *a rule by which the doses of all homœopathic medicines, without exception, are to be attenuated to such a degree that after being introduced into the body they shall produce an almost insensible aggravation of the disease.*"

(By the way, this "incontrovertible axiom" is an *ex post facto* guide after all, for, of course, you cannot detect an aggravation until after your remedy has been administered, and in this case it ceases to be a guide.)

With all due deference to the illustrous founder of Homœopathy, it seems questionable to us whether a cure always and necessarily depends upon a perceptible aggravation of the symptoms. Indeed, we believe it to be within the experience of every careful observer to meet with cases where relief and nothing but relief has promptly succeeded the administration of the well-selected homœopathic remedy. Relief sometimes so speedy that it seemed almost like magic.

We often get aggravations, to be sure, and in such cases are taught to regard the aggravation as an indication that the remedy has been well chosen, but has either been given in too low a potency or else has been repeated too often.

Some of our friends assert that aggravations are more likely to follow the injudicious use of the highest rather than of the lower potencies. While I do not positively dispute this assertion, I must say that I am not yet convinced of its truth.

In discussing the law of dose, Dr. Wells has told us that an

"Explanation is found in the degree of susceptibility of the patient to the action of the drug, and this in the direct ratio of the similarity of the characteristics of the drug and the disease." (*Am. Hom. Review*, vol. V, p. 251.)

Dr. Fincke gives us the same idea in different language, as follows: "The relation of the remedy to the life-force demands its proper selection according to simility of symptoms and dose."

In further elucidation of this subject, Dr. Fincke asks and replies to the following question, viz.:

"Which will be the appropriate potency in the given case?"

Answer—"That one which will be proportionate to the degree of susceptibility of the patient." (*Journ. of Hom.*, vol. II, p. 288.)

Theoretically the above rules are philosophical and sound, but do they greatly aid the practitioner in the choice of the exact potency?

The practitioner may understand that the closer the resemblance between disease and drug symptoms the greater is the susceptibility of the patient, and consequently the higher is the potency required, but the whole problem being one of comparative relations, still demands the exercise of individual judgment.

It being granted that the susceptibility of the patient is in ratio to the simility of symptoms, we must constantly meet with cases where these comparative relations differ in degree—*i. e.*, are more or less alike. Does it not then logically follow that in order to select the appropriate curative we are bound to exercise our individual judgments, which may lead us either up or down the scale of potencies?

Personally, I do not regard the use of the lower potencies, provided they are used strictly in subordination to the rules (as, unfortunately, is scarcely ever done when the lower potencies are exclusively employed). I say I do not regard the use of low potencies as absolutely and necessarily unhomœopathic, but as a very questionable, crude, and decidedly inferior kind of Homœopathy.

We never need despair for the ultimate advancement of the practitioner who carefully individualizes his cases and endeavors to exercise a wise discretion in the matter of dose, for that practitioner is on the highway toward the Hahnemannian standing-point, and deserves our encouragement for his good intentions, instead of our denunciations for what we may regard his short-comings.

In conclusion, notwithstanding some minor differences of opinion, let us extend the right hand of fellowship to every practitioner who adheres faithfully to the law of similars, and who confines his practice to the single remedy used in what he thoughtfully conceives to be the minimum dose.

Adjourned to 8 P. M.

